# Antimicrobial Activity of Artemisinin and Precursor Derived from *In Vitro* Plantlets of *Artemisia annua* L.

**DOI:** 10.1155/2014/215872

**Published:** 2014-01-19

**Authors:** Suganthi Appalasamy, Kiah Yann Lo, Song Jin Ch'ng, Ku Nornadia, Ahmad Sofiman Othman, Lai-Keng Chan

**Affiliations:** Plant Tissue and Cell Culture Laboratory, School of Biological Sciences, Universiti Sains Malaysia, 11800 Penang, Malaysia

## Abstract

*Artemisia annua* L., a medicinal herb, produces secondary metabolites with antimicrobial property. In Malaysia due to the tropical hot climate, *A. annua* could not be planted for production of artemisinin, the main bioactive compound. In this study, the leaves of three *in vitro A. annua* L. clones were, extracted and two bioactive compounds, artemisinin and a precursor, were isolated by thin layer chromatography. These compounds were found to be effective in inhibiting the growth of Gram-positive and Gram-negative bacteria but not *Candida albicans*. Their antimicrobial activity was similar to that of antibactericidal antibiotic streptomycin. They were found to inhibit the growth of the tested microbes at the minimum inhibition concentration of 0.09 mg/mL, and toxicity test using brine shrimp showed that even the low concentration of 0.09 mg/mL was very lethal towards the brine shrimps with 100% mortality rate. This study hence indicated that *in vitro* cultured plantlets of *A. annua* can be used as the alternative method for production of artemisinin and its precursor with antimicrobial activities.

## 1. Introduction

*Artemisia annua* L., an annual medicinal herb, can be found growing wild in the temperate and high altitude regions of China and Vietnam [1, 2]. Traditionally it is used to alleviate high fever and treatment of jaundice [3]. Artemisinin, one of the bioactive compounds, with antimalarial activity has been successfully isolated from *A. annua* [4]. Other than antimalarial activity, artemisinin was found to be a good antibacterial, antifungal, antileishmanial, and antitumor agent. The antibacterial properties of artemisinin had been tested on a wide range of bacteria, such as *Escherichia coli *[5], *Staphylococcus aureus*, *Pseudomonas aeruginosa,* and *Mycobacterium intracellulare* [6]. A broad spectrum of other secondary metabolites was found and accumulated at the aerial part of *A. annua*. However, the secondary metabolite contents are often influenced by environmental stresses [7, 8]. In Malaysia, the hot tropical weather delimits the planting of this herb as crop plant, and thus *in vitro* culture technique can be used as the alternative tool for the production of artemisinin. However, secondary metabolites that are produced *in vitro* often differ in type and amount than those produced in field cultivated plants due to biotic and abiotic stresses [9, 10]. The focus of this paper was hence to report whether the bioactive compounds derived from the leaves of *in vitro* plantlets of *A. annua *possess antimicrobial activity towards an array of bacteria and fungus of Malaysian local isolates and also the toxicity level of these compounds on brine shrimp. These toxicity assays [11] are used to assess the toxicity level of the bioactive compounds derived from the *in vitro *plantlets of *A. annua*.

## 2. Materials and Methods

### 2.1. Plant Material

Three different clones of *A. annua* L. of Vietnam origin, TC1, TC2, and Highland, were established from seeds and cultured on MS [12] medium. The excised nodal segments from the eight weeks old seed-derived *in vitro* plantlets were subsequently cultured on MS basal medium containing 30 g/L sucrose and 8 g of Agar (Algas, Chile) for mass production of plant materials for the present study. The *in vitro* plantlets were maintained under a constant temperature of 25 ± 2°C with continuous lighting of approximately 32.5 *μ*mol m^−2^ s^−1^ light intensity. The pH of all the culture media used in this study was adjusted to pH 5.7–5.8 before autoclaving (Tommy 325) at 121°C for 11 minutes under 1.05 kg/cm^2^ pressure. Harvested plantlets were air dried at room temperature until constant dried weight was obtained.

### 2.2. Extraction and Fraction of Crude Extract

Dried aerial parts (20 g) of the three different clones cultured on the MS [12] medium were powdered with mortar and pestle. They were extracted with *n*-hexane (AR grade) with the aid of ultrasonication. The collected supernatants were evaporated into dry extract using rotary evaporator. The crude extracts were dissolved in a combination of acetonitrile (Sigma) and *n*-hexane (Sigma) solvents and partitioned using a separation funnel. The partitioned parts of solvents were tested for artemisinin using thin layer chromatography (TLC). The fraction with artemisinin was dried using rotary evaporator. Then, the dried fraction was weighed and purified via column chromatography based on the method by El-Feraly et al. [13]. Fractions of 1 mL were tested for presence of artemisinin, and fractions that contained artemisinin and a precursor located very near to artemisinin (tested via TLC) were then pooled together and dried with rotary evaporator. It was then purified again by eluting in column chromatography as mentioned above. Fractions with artemisinin and a precursor were pooled into a flask, respectively, and weighed.

### 2.3. Preparation of Bacterial and Fungal Cultures

Three Gram-positive USM bacteria strains, *Staphylococcus aureus, Bacillus thuringiensis,* and* Bacillus subtilis, *two Gram-negative USM bacteria strains,* Escherichia coli *and *Salmonella *sp., and *Candida albicans* (yeast, USM strain) were used for antimicrobial activities studies. The bacterial strains were grown in Nutrient Agar (NA) plates and the yeast was grown in Sabouraud Dextrose Agar (SDA) medium. All microbial cultures were incubated at 37°C while the stock cultures were maintained at 4°C.

### 2.4. Evaluation of Antimicrobial Activities

#### 2.4.1. Antimicrobial Disk Diffusion Assay

Nutrient Agar (NA) and Sabouraud Dextrose Agar (SDA) were prepared and sterilized in a Schott bottle and cooled before poured into sterilized petri dishes (diameter 9 cm). The bacteria and yeast were then cultured on the solid plates with sterile cotton bud. The filter paper (Whatman) discs with the diameter of 0.6 cm were placed on the agar plates cultured with the tested microorganisms. Filter paper discs impregnated with 1 *μ*L of acetonitrile and streptomycin were used as negative and positive controls, respectively. Purified extracts were impregnated on the filter paper discs accordingly. All the plates were incubated at 37°C for 48 h. The diameters of the inhibition zones were measured every six hours during the 48 h incubation period. All the tests were performed in triplicate.

#### 2.4.2. Minimum Inhibition Concentration (MIC) Measurement

Minimum inhibition concentration (MIC) for each microbe was determined based on the least concentrations of artemisinin and precursor needed to inhibit the growth of the tested microbes. A serial dilution of artemisinin and precursors was done so that the concentration of the artemisinin and precursor was in range of 0.09 mg/ml to 3 mg/ml. Six disks of all the six concentrations were impregnated on each plate of tested microbes. The test was done in triplicates for each compound derived from each clone.

#### 2.4.3. Toxicity Test for Artemisinin and Precursor

Lethal concentration 50 (LC_50_) is the measurement of the concentration of an extract that kills half of the sampling population. The two fractions of compounds (artemisinin and precursor) obtained from the three clones were tested against brine shrimps (*Artemia salina*). Brine shrimp was prepared by hatching 50 mg of eggs in artificial sea water (30 g/L NaCl). The brine shrimp eggs were placed under constant lighting for 24 hours. A serial dilution of the compounds was done so that the concentration of the compounds was in range of 0.09 mg/mL to 3 mg/mL. The diluted compounds were then transferred into 96-well microtiter plate. Ten brine shrimps were loaded into each well containing the compounds. The experiment was done in six replicates for each dilution factor of a compound. The brine shrimps were incubated under constant light at 30°C for 24 hours. Artificial seawater was used as control for each compound.

## 3. Results

### 3.1. Extraction of Artemisinin and Precursor from *In Vitro A. annua *L. plantlets

**Figure 1:**
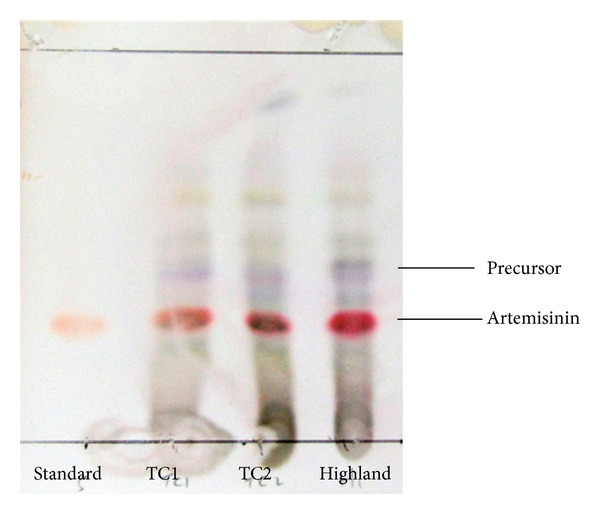
Thin layer chromatography (TLC). Purple band denotes precursor and pink band denotes artemisinin compounds which were purified separately by column chromatography and used for the antimicrobial screening and toxicity test.

The amount of crude extract obtained from 20 g dried leaves of *A. annua* was found to be different for each clone. The highest yield of crude extract could be obtained from TC2 clone followed by the Highland and TC1 clones. The crude extracts were then fractioned and purified by column chromatography. The results of column chromatography purification indicated that all the three tested clones of *in vitro A. annua* plantlets contained between 2.90 and 3.75 mg/g of artemisinin with Highland clone (3.75 mg/g) and TC2 clone (3.55 mg/g) produced higher artemisinin as compared to TC1 clone. Whereas the content of precursor in the three clones of *A. annua in vitro* plantlets was in the range of 1.85 and 3.9 mg/g with TC2 clone produced the highest precursor content (3.9 mg/g) followed by TC1 clone (2.3 mg/g) and the Highland (1.85 mg/g) (Table 1). These two compounds were identified and distinguished from each other using thin layer chromatography (TLC) through the comparison with artemisinin standard (98% purity, Sigma). The precursor above artemisinin which could be an artemisinin derivative was clearly separated from artemisinin and very visible in all the extracts from the three *in vitro* clones (Figure 1). These two compounds obtained from each *A. annua *clone were used for the subsequent antimicrobial screening and toxicity tests.

### 3.2. Evaluation of Antimicrobial Effect of Artemisinin and Precursor and Determination of MIC Value

A preliminary antimicrobial screening test using disk diffusion technique was done on locally isolated six microorganisms consisted of Gram-positive and negative strains bacteria and one fungus. Artemisinin and precursor were tested on three Gram-positive strains, *Staphylococcus aureus, Bacillus thuringiensis,* and *Bacillus subtilis,* two Gram-negative strains, *Escherichia coli* and *Salmonella* sp., and a yeast strain, *Candida albicans*. Among all the tested microbes, artemisinin of the three *A. annua* clones was most effective on *S. aureus* with TC2 and Highland having the same inhibition zone (3 ± 1.58 mm) as that of streptomycin (positive control). TC1 clone which has inhibition zone of 2 ± 1.15 mm was not significantly different from the positive control. This indicated that artemisinin could be an effective anti-*S. aureus* drug.* B. subtilis* and *B. thuringiensis* showed inhibition zone of 1 ± 0.00 mm when treated with artemisinin derived from the three clones. This also showed that artemisinin could be an antimicrobial drug against Gram-positive bacteria. Between the two tested Gram-negative strains, only *Salmonella *sp.,  showed inhibition growth due to artemisinin derived from the three clones, and their anti-Salmonella activities were similar to that of streptomycin, the positive control. Artemisinin from the three clones did not exhibit any antimicrobial activity on *E. coli *and *C. albicans* (Table 2).

Precursor from all the three clones showed antimicrobial effect towards both the Gram-positive and Gram-negative bacteria except the yeast, *C. albicans*. Precursor derived from TC1 showed the strongest effect on *E. coli*, and this was not significantly different from that of streptomycin, the positive control. The anti-*E. coli* activity was in the order of TC1 > TC2 > Highland. This indicated that precursors from the three clones were effective as anti-bacteria for both Gram-positive and Gram-negative. On the other hand, precursor did not inhibit the growth of *C. albicans* (Table 3).

From this preliminary antimicrobial assay, the growth of the three bacteria strains (*B. subtilis, S. aureus, *and *Salmonella *sp.) was inhibited by both artemisinin and its precursor; hence they were chosen for the minimum inhibitory concentration (MIC) assay. MIC assay was done to determine the lowest concentration of compounds that inhibits the microbial growth. The result of MIC on the three tested microbes indicated that the lowest concentration of both artemisinin and its precursor derived from the three clones, TC1, TC2, and Highland was, 0.09 mg/mL which was effective to inhibit all the growth of the three tested microbes (Table 4).

### 3.3. Toxicity Study of Artemisinin and Precursor

Toxicity test of artemisinin and precursor from the three *in vitro A. annua* L. clones on brine shrimp showed that inhibition of brine shrimp growth still occurred even at the lowest tested concentration (0.09 mg/mL) of the compounds. Within one hour of incubation, the brine shrimps were all dead indicating high toxicity level of artemisinin and precursor against brine shrimp growth, and thus LC_50_ could not be determined.

## 4. Discussion

The antimicrobial effects of artemisinin and precursor extracted from *in vitro *plantlets of *A. annua* were tested on the chosen six microbes which causes illness in human [14–16]. Results obtained indicated that artemisinin and its precursor were effective against Gram-positive bacteria, and their antibacterial activities were similar to that of streptomycin, a bactericidal antibiotic. Plant extracts from Asteraceae family against Gram-positive strain bacteria had been reported previously [17–21]. Artemisinin derived from field grown *A. annua *plants was also reported to have antimicrobial activity [22–24]. The susceptibility activity of Gram-positive strains to artemisinin and precursor derived from *in vitro A. annua *plantlets which had not been reported before confirmed that the *in vitro* plantlets could produce bioactive compounds that were similar to that found in the field grown mature plants of *A. annua. *These artemisinin and precursor produced from the *in vitro* plantlets also possess antimicrobial activity comparable to streptomycin. Hence, the present study indicated that the *in vitro* plantlets of *A. annua *could be used as an alternative mean for the production of artemisinin and its precursor in tropical countries like Malaysia as *A. annua *cannot be grown in the constantly hot tropical weather [25]. Moreover, the artemisinin and its precursor produced from the *in vitro* plantlets are effective towards the Gram-positive strains bacteria at a low concentration (0.09 mg/mL) as indicated by the MIC results. The susceptibility of Gram-positive strains towards photochemical compounds derived from *A. annua* was caused by the inhibition of the efflux pump in the bacteria [26].

Gram-negative strains used in this study were *Salmonella *sp. and *E. coli*. The artemisinin derived from the *in vitro* plantlets of *A. annua *was only effective for *Salmonella *sp. but not the *E. coli*. However the precursor was effective against both Gram-negative bacteria especially the *E. coli*. The resistance of Gram-negative strain towards artemisinin suggested that these bacteria have multidrug resistance due to the presence of active multiefflux pumps. This active multiefflux pump of inhibitory substance is a very important part of the antimicrobial compound defence in bacteria [27]. The permeability of cell walls of Gram-negative and Gram-positive bacteria differs greatly in terms of the rate of large molecules penetrations [28]. This was one of the reasons Gram-negative bacteria were more resistant to antimicrobial compounds which supported the findings of this study. However, the precursor in this study was found to be more effective in growth inhibition of *E. coli* bacteria compared to artemisinin. Isolated plant compounds which reported to have antibacterial property against Gram-positive strains normally do not work likewise for Gram-negative strain [29]. The susceptibility of *E. coli *to the precursor derived from the *A. annua in vitro* plantlets suggested that this compound was coextracted with fatty acids which successfully inhibited the efflux pumps in *E. coli* [30]. The result obtained from this study further confirmed the inability of artemisinin and precursor to inhibit *C. albicans* as reported by Galal et al. [22] that artemisinin and its derivatives were not effective for inhibiting the growth of *C. albicans* and *Cryptococcus neoformans*.

Minimum inhibitory concentration (MIC) value for both artemisinin and its precursor derived from the *in vitro* plantlets of three *A. annua *clones showed that a very low concentration (0.09 mg/mL) was sufficient to inhibit the growth of *Bacillus subtilis* and *Staphylococcus aureus* (Gram-positive bacteria) and *Salmonella *sp. (Gram-negative bacteria). Nagshetty et al. [31] reported that three antibiotics, Nalidixic acid, Ampicillin, and Chloramphenicol, had MIC values in the range of 32–256 *μ*g/mL while the MIC value for Ciprofloxacin was achieved in the range of 0.125–4 *μ*g/mL towards *Salmonella typhi*. This indicated that different antibiotics have different antimicrobial capability. Some require much higher concentration whereas very low concentration of Ciprofloxacin, normally used in very purified form, was needed to inhibit the growth of *S. typhi* when compared to the artemisinin and precursor (90 *μ*g/mL) derived from the tissue cultured plantlets of *A. annua* used in this study. While artemisinin of 9 mg/mL derived from the field grown plants was needed to inhibit malaria causing *Plasmodium falciparum* [32]. The result obtained from our study on the brine shrimp toxicity test suggested that artemisinin and precursor could be very toxic when used at high concentration because as low as 0.09 mg/mL of both the artemisinin and its precursor caused high mortality rate (100%) of the brine shrimp.

## 5. Conclusion

The antimicrobial activity of artemisinin and precursor derived from the *in vitro *plantlets of *A. annua *was found to be having antimicrobial capability as that of the commercially available antibiotic, streptomycin. The ability to inhibit microbial growth suggested that *in vitro* cultured *A. annua *could be an alternative for the production of these potential antimicrobial drugs.

## Figures and Tables

**Table 1:** Yield of crude extract, artemisinin, and precursor from the dried leaves of three clones of *A. annua*.

*A. annua* clone	Crude extract (mg/g)	Artemisinin (mg/g)	Precursor (mg/g)
TC1	16.65	2.90	2.30
TC2	19.70	3.55	3.90
Highland	17.90	3.75	1.85

**Table 2:** Antimicrobial activity of artemisinin (6 mg/mL) isolated from three clones of *A. annua *L., streptomycin (6 mg/mL) as positive control and acetonitrile as negative control tested by disk diffusion assay.

Microorganisms	Inhibition zone (mm)				
	Artemisinin			Control	
	TC1	TC2	Highland	Streptomycin (positive )	Acetonitrile (negative)
*Bacillus subtilis *	1 ± 0.41^a^	1 ± 0.82^a^	1 ± 0.82^a^	1 ± 0.41^a^	0 ± 0.00^b^
*Staphylococcus aureus *	2 ± 1.15^a^	3 ± 1.58^a^	3 ± 1.58^a^	3 ± 2.24^a^	0 ± 0.00^b^
*Bacillus thuringiensis *	1 ± 0.00^a^	1 ± 0.00^a^	1 ± 0.00^a^	1 ± 0.00^a^	0 ± 0.00^b^
*Escherichia coli *	0 ± 0.00^b^	0 ± 0.00^b^	0 ± 0.00^b^	3 ± 0.00^a^	0 ± 0.00^b^
*Salmonella *sp.	1 ± 0.00^a^	2 ± 1.29^a^	1 ± 0.00^a^	1 ± 0.00^a^	0 ± 0.00^b^
*Candida albicans *	0 ± 0.00^b^	0 ± 0.00^b^	0 ± 0.00^b^	10 ± 0.82^a^	0 ± 0.00^b^

Values are mean inhibition zone (mm) ± SD of three replicates.

Mean values of inhibition zones of each microorganism followed by the same alphabet were not significantly different (Tukey test, *P* ≤ 0.05).

**Table 3:** Antimicrobial activity of precursor (6 mg/mL) isolated from three clones of *A. annua *L*.,* streptomycin (6 mg/mL) as positive control and acetonitrile as negative control tested by disk diffusion assay.

Microorganisms	Inhibition zone (mm)				
	Precursor			Control	
	TC1	TC2	Highland	Positive	Negative
*Bacillus subtilis *	1 ± 0.89^a^	1 ± 0.63^a^	1 ± 0.63^a^	1 ± 2.23^a^	0 ± 0.00^b^
*Staphylococcus aureus *	3 ± 2.41^a^	2 ± 1.18^a^	3 ± 1.40^a^	3 ± 2.28^a^	0 ± 0.00^b^
*Bacillus thuringiensis *	1 ± 0.00^a^	1 ± 0.00^a^	1 ± 0.0^a^	1 ± 0.58^a^	0 ± 0.00^b^
*Escherichia coli *	3 ± 0.00^a^	2 ± 0.00^b^	1 ± 0.00^c^	3 ± 0.00^a^	0 ± 0.00^d^
*Salmonella spp. *	1 ± 0.00^a^	1 ± 0.50^a^	1 ± 0.50^a^	1 ± 0.00^a^	0 ± 0.00^b^
*Candida albicans *	0 ± 0.00^b^	0 ± 0.00^b^	0 ± 0.00^b^	10 ± 1.08^a^	0 ± 0.00^b^

Values are mean inhibition zone (mm) ± SD of three replicates.

Mean values of inhibition zones of each microorganism followed by the same alphabet were not significantly different (Tukey test, *P* ≤ 0.05).

**Table 4:** Minimum inhibitory concentration (MIC) value of artemisinin and its precursor derived from the three *A. annua *clones on selected microorganism.

Microorganisms	Minimum inhibition concentration (MIC) in mg/mL					
	TC1 clone		TC2 clone		Highland clone	
	Precursor	Artemisinin	Precursor	Artemisinin	Precursor	Artemisinin
*Bacillus subtilis *	0.09	0.09	0.09	0.09	0.09	0.09
*Staphylococcus aureus *	0.09	0.09	0.09	0.09	0.09	0.09
*Salmonella *sp.	0.09	0.09	0.09	0.09	0.09	0.09
